# Impact of Site of Care on Infection Rates Among Patients with Primary Immunodeficiency Diseases Receiving Intravenous Immunoglobulin Therapy

**DOI:** 10.1007/s10875-017-0371-0

**Published:** 2017-02-03

**Authors:** Richard L. Wasserman, Diane Ito, Yan Xiong, Xiaolan Ye, Patrick Bonnet, Josephine Li-McLeod

**Affiliations:** 1 Allergy Partners of North Texas, 7777 Forest Lane, Suite B-332, Dallas, TX 75230 USA; 2 Health Economics, Outcomes Research & Epidemiology, Shire, 650 Kendall St, Cambridge, MA 02142 USA

**Keywords:** Immunoglobulin G, Primary immunodeficiency, Infection, Clinic, Hospital outpatient, Home infusion

## Abstract

**Purpose:**

Patients with primary immunodeficiency diseases (PIDD) are at increased risk of infection and may require lifelong immunoglobulin G (IgG) replacement. Infection incidence rates were determined for patients with PIDD receiving intravenously administered IgG (IGIV) in a home or hospital outpatient infusion center (HOIC).

**Methods:**

Data were extracted from a large, US-based, employer-sponsored administrative database. Patients were eligible for analysis if they had ≥1 inpatient or emergency room claim or ≥2 outpatient claims with a PIDD diagnosis between January 2002 and March 2013, 12 months of continuous health plan enrollment prior to index date (i.e., first IGIV infusion date), and 6 months of continuous IGIV at the same site of care after the index date. Incidences of pneumonia (bacterial or viral) and bronchitis (all types) within 7 days of IGIV infusion were retrospectively determined and compared between sites of care.

**Results:**

A total of 1076 patients were included in the analysis; 51 and 49% received IGIV at home and at an HOIC, respectively. The event/patient-year of pneumonia was significantly lower in patients receiving IGIV at home compared to an outpatient hospital (0.102 vs. 0.216, *p* = 0.0071). Similarly, the event/patient-year of bronchitis was significantly lower among patients infusing at home compared to an HOIC (0.150 vs. 0.288, *p* < 0.0001).

**Conclusions:**

PIDD patients experienced incidence rates for pneumonia and bronchitis that were lower for patients receiving home-based IGIV treatment versus HOIC-based IGIV treatment. The lower infection rates in the home setting suggest that infection risk may be an important factor in site of care selection.

## Introduction

Primary immunodeficiency diseases (PIDDs) are a heterogeneous group of more than 250 congenital disorders characterized by aberrant immune responses [1]. PIDDs resulting from primary antibody deficiencies, which account for the majority of PIDD diagnoses, leave patients susceptible to repeated and serious infections, particularly those of the respiratory tract [1].

Human immunoglobulin G (IgG) replacement constitutes the foundation of treatment for PIDD patients with disorders of antibody production [2–4]. Many studies have established an inverse relationship between trough serum levels of IgG and the incidence of infections [5–10]. The United States (US) Food and Drug Administration has approved the administration of IgG intravenously (IGIV) and subcutaneously (IGSC) for multiple indications, including the treatment of patients with PIDD [4]. In patients with PIDD, the clinical benefits of IgG replacement include decreased risk of upper and lower respiratory tract bacterial infections resulting in a concomitant reduction in antibiotic use and hospitalizations as well as improvement in pulmonary function and quality of life [5–7, 11].

Ideal care of PIDD patients requires optimal dosing of IgG [5, 6, 8–11]. However, IgG replacement is a long-term treatment that exacts a significant time commitment from patients. IGIV infusions are generally administered every 3 to 4 weeks over a median duration of 2.3 h [12–15]. Conventional IGSC infusions are administered weekly over a median duration of 1.2 h [10, 16], while human hyaluronidase-facilitated subcutaneous IgG (HyQvia [IGHy]; Baxalta US Inc., Westlake Village, CA) [17] infusions average 2.08 h every 3 to 4 weeks [14]. An estimated 40% of patients with PIDD need to incorporate similar dosing regimens for the duration of their lives [2, 16]. Travel time to appointments is an additional patient burden, which can negatively impact adherence [18].

For PIDD patients in the USA, IgG infusions may be administered at different sites of care, including a physician’s office, a hospital outpatient infusion center, a freestanding infusion center, or a patient’s home. The safety profile of IGIV in patients with PIDD has been well documented [9, 19], and home-based IGIV infusions have become relatively commonplace in this population [18], as well as in other patient populations requiring chronic IgG treatment [20]. Home administration of IGIV is not only more convenient for patients and their families but also potentially decreases exposure to pathogens that may be prevalent in other settings of care [21]. This is clinically relevant for patients with PIDD who already have increased susceptibility to infections [1, 3]. Additional data shows improved patient adherence and significantly lower cost of care for patients treated with IGIV at home compared with a hospital outpatient infusion center setting [18].

While home-based infusion offers advantages compared with infusions administered at other sites of care, to the authors’ knowledge, there is no documentation in the literature explaining how site of care impacts the incidence of infections in patients with PIDD. A retrospective analysis was therefore undertaken to determine the rates of pneumonia and bronchitis among patients with PIDD treated with IGIV in a hospital outpatient infusion center- or home-based setting.

## Methods

### Database Description

This retrospective analysis utilized a large administrative claims database (years 2002–2013) which consists of fully adjudicated and paid claims, integrated enrollment, inpatient, outpatient, and drug data from US-based employer-sponsored commercial and Medicare supplemental plans. This database represents over 50 million covered lives.

### Patient Population

Patients who had at least one inpatient or emergency room claim or at least two outpatient claims with a diagnosis for PIDD (International Classification of Disease Codes [ICD-9] diagnosis of 279.xx) between January 1, 2002 and March 31, 2013 were identified from the database. The ICD-9 code 279.xx is a root code for PIDDs [22]. Eligible patients had at least 12 months of continuous health plan coverage prior to their first IGIV infusion, which served as their index date. Patients also received at least 6 months of continuous IGIV at the same site of care (home or hospital outpatient infusion center) after their index date to be included in the study.

### Statistical Analysis

#### Number of Infections and Incidence Rates

The number of infections and incidence rates of pneumonia (bacterial and viral) and bronchitis (including acute, chronic, and not otherwise specified) were calculated for each site of care. Pneumonia was defined using ICD-9 diagnosis codes 481, 482, 483, 486, and v12.61, while bronchitis was defined using diagnosis codes 466 (acute), 491 (chronic), and 490 (not specified). Infections that occurred within 4 weeks of an IGIV infusion were captured. In addition, an infection episode was defined as a ≤7-day gap in claims for a particular infection rate diagnosis. If the same diagnosis codes for the infection occurred within 7 days, it was considered as one infection episode.

Generalized linear model regression analyses were conducted to compare age-, gender-, and illness severity-adjusted infection rates between sites of care. Specifically, Poisson regression models using log link and Poisson distributions were used to compare the rate of infection episodes (pneumonia; bronchitis) between sites of care. Separate regression analyses were run for each of the 4 weeks post-infusion to assess the impact of time from infusion on infection rates between sites of care. Covariates in the regression models included the following variables: age, sex, Charlson Comorbidity Index (CCI), and prophylactic antibiotic use, infection rates, emergency room, inpatient and outpatient visits in the 12 months prior to IGIV use. To control for illness severity between the patients in each site of care, several variables were identified and used as proxies for illness severity, including CCI score, prophylactic antibiotic use, infection rate, inpatient visits, and outpatient visits in the past 12 months prior to IGIV use. Infection rates occurring in the past 12 months prior to the first IGIV use included the following types of infections: pneumonia, bronchitis, otitis media, meningitis, cold, sinusitis, pharyngitis, laryngitis, respiratory, tonsillitis, bronchiectasis, diarrhea, or sepsis. SAS version 9.3 [23] (SAS Institute, Inc., Cary, NC, USA) was used to perform statistical analyses with a priori significance level of 0.05.

## Results

### Patient Characteristics

A total of 1076 PIDD patients were identified for the current analysis **(**Table 1 **)**. Fifty-one percent of patients received IGIV in a home setting. The most frequent ICD-9 code diagnosis was CVID for both home (51.7%) and hospital outpatient infusion center (44.0%) settings. Patients receiving IGIV infusions in the home setting were generally younger than those receiving infusions in the hospital outpatient infusion center setting, with a mean age of 45 (range 1–88) and 54 (range 1–90) years, respectively. Prophylactic antibiotic use was more frequent in patients receiving home treatment (59.4%) compared with patients infused in an outpatient hospital (48.1%). Additionally, the proportion of patients infusing in a home setting who had CCI scores of ≥2 was significantly lower compared to patients infusing in a hospital outpatient infusion center setting (34 vs. 52%, respectively, *p* < .05), and the proportion of patients with ≥1 inpatient visits 12 months prior to IGIV use was also significantly lower among patients infusing at home compared to the hospital outpatient infusion center (27 vs. 40%, *p* < 0.05) (see Table 1). This suggested that patients infusing in the home setting, in addition to being younger, were healthier overall, but received more prophylactic antibiotics, compared to those who infused in the hospital outpatient infusion center setting. To control for these differences, these variables were included as covariates in the regression analysis.

**Table 1:** Patient characteristics

Patient characteristic	Site of care	
	Home	Hospital outpatient infusion center
Total patients, *n*	544 (50.6)	532 (49.4)
Sex, *n* (%)		
Female	368 (67.6)	332 (62.4)
Male	176 (32.4)	200 (37.6)
Age, years, mean (min–max)*	45.3 (1–88)	53.5 (1–90)
Diagnosis (ICD-9 Codes) [29], *n* (%)		
CVID (279.06)	281 (51.7)	234 (44.0)
Hypogammaglobulinemia (279.00)	102 (18.8)	159 (29.9)
Other immune mechanism disorders (279)	85 (15.6)	85 (16.0)
Selective immunoglobulin deficiencies (279.03)	51 (9.4)	36 (6.8)
XLA (279.04)	16 (2.9)	7 (1.3)
SAD (279.01)	4 (1.0)	8 (1.5)
Hyper IgM (279.05)	5 (1.0)	3 (1.0)
CCI score, *n* (%)*		
0	158 (29)	94 (17.7)
1	202 (37)	163 (30.6)
2	83 (15.3)	106 (19.9)
≥3	101 (18.6)	169 (31.8)
Infections within 12 months of IGIV initiation, *n* (%)^a^		
0	89 (16.4)	85 (16.0)
1–4	194 (35.7)	208 (39.1)
5–8	118 (21.7)	120 (22.6)
≥9	143 (26.3)	119 (22.4)
Inpatient visits within 12 months of IGIV initiation, *n* (%)*		
0	398 (73.2)	322 (60.5)
1	94 (17.3)	116 (21.8)
≥2	52 (9.6)	94 (17.7)
Outpatient service^b^ within 12 months of IGIV initiation, *n* (%)		
1–24	151 (27.8)	159 (29.9)
25–36	131 (24.1)	126 (23.7)
37–60	156 (28.7)	145 (27.3)
≥61	106 (19.5)	102 (19.2)
ER visits within 12 months of IGIV initiation, *n* (%)		
0	381 (70.0)	349 (65.6)
1	91 (16.7)	92 (17.3)
≥2	72 (13.2)	91 (17.1)
Prophylactic antibiotic use within 12 months pre-index date, *n* (%)	323 (59.4)	256 (48.1)

*CCI* Charlson Comorbidity Index, *CVID* common variable immunodeficiency, *ER* emergency room, *ICD* International Classification of Disease Codes, *IGIV* intravenously administered IgG, *IgM* immunoglobulin M, *SAD* selective antibody deficiency, *XLA* X-linked agammaglobulinemia (Bruton’s agammaglobulinemia)

*Significant difference between sites of care, *p* < 0.05

^a^Infections within the 12 months prior to IGIV use included: pneumonia (bacterial or viral), any bronchitis, otitis media, meningitis, encephalitis, influenza, acute or chronic sinusitis, acute or chronic pharyngitis, laryngitis, respiratory illness, tonsillitis, bronchiectasis, diarrhea, or sepsis

^b^Outpatient service may include outpatient hospital, clinic, hospice, or other facility in which an outpatient service was performed. Numbers represent a unique date of service

### Infection Episodes and Incidence Rates

Among the 1076 patients included in this analysis, 581 episodes of infection (pneumonia, *n* = 244; bronchitis, *n* = 337) were identified (Table 2). After adjusting for age, sex, Charlson comorbidities, infection rates, and prophylactic antibiotic use, emergency room, inpatient and outpatient visits in the 12 months prior to IGIV use, incidences of infections (rates per patient-year) were significantly lower among patients who received IGIV infusions in the home compared with a hospital outpatient infusion center setting (see Table 2). Patients who received IGIV in a hospital outpatient infusion center were 1.5 times more likely to develop bronchitis and 1.8 times more likely to develop pneumonia compared with those who received treatment in a home setting.

**Table 2:** Number and infection rates of pneumonia and bronchitis (occurring within 7 days of IGIV infusion) by site of care

Infection type	Site of care (1076 patients)				Difference between hospital outpatient infusion center and home, *p* value
	Home (544 patients)		Hospital outpatient infusion center (532 patients)		
	Episodes^a^, *n*	Infection rate per pt-yr^b^	Episodes^a^, *n*	Infection rate per pt-yr^b^	
Pneumonia^c^ (244 episodes)	85	0.102	159	0.216	0.0071
Bronchitis^d^ (337 episodes)	125	0.150	212	0.288	<0.0001

*pt-yr* patient-year

^a^An infection episode was defined as ≤7-day gaps in claims for a particular infection rate diagnosis

^b^Pt-yrs = 834.9 (home) and 736.3 (hospital outpatient infusion center)

^c^Bacterial or viral

^d^Any type of bronchitis

Additional analyses were undertaken to examine if the time interval post-infusion had an impact on infection rates between sites of care (Table 3). Overall, pneumonia and bronchitis were most frequent in the first week post-infusion and declined thereafter. Pneumonia patients who infused in an outpatient hospital setting, compared with home-based infusion, had significantly higher rates of infection in the first and third week after infusion (*p* = 0.0004 and *p* = 0.0265, respectively), but not in the second and fourth week (*p* = 0.073 and 0.533, respectively). Bronchitis patients who infused in the outpatient hospital setting had significantly higher rates of infection in the first and second week post-infusion (*p* < 0.0001 and *p* = 0.005, respectively), but did not differ significantly in the third or fourth week (*p* = 0.477 and *p* = 0.597, respectively), compared with home-based infusions (see Table 3).

**Table 3:** Number and infection rates (unadjusted) of pneumonia and bronchitis per person-week by post-infusion time interval

Infection type, by post-infusion week	Unadjusted episodes each week, *n*		Unadjusted infection rates per person-week		Point estimate outpatient vs. patient home	*p* value^a^
	Home (544 patients)	Hospital outpatient infusion center (532 patients)	Patient home	Outpatient hospital		
Pneumonia						
Week 1	85	159	0.0073	0.0181	0.5137	0.0004
Week 2	26	33	0.0025	0.0038	0.3378	0.0734
Week 3	41	79	0.0042	0.0096	0.4461	0.0265
Week 4	46	69	0.0060	0.0096	0.1218	0.5330
Bronchitis						
Week 1	125	212	0.0107	0.0241	0.6563	<0.0001
Week 2	51	79	0.0049	0.0091	0.4228	0.0055
Week 3	95	119	0.0097	0.0144	0.1034	0.4733
Week 4	93	108	0.0121	0.0151	0.0778	0.5973

^a^ *P* values reflect adjusted values generated from the generalized linear model regression analyses

## Discussion

Site of care for administration of infusions has been shown to have an impact on patients and their caregivers by affecting sense of control, ability to maintain normal daily activities (e.g., work, school, time with family), cost of care, treatment adherence, and risk of infection, regardless of disease state [16, 18, 24–26]. Treatment for patients with PIDD can be administered at different sites of care, including at a hospital outpatient infusion center or at home. Studies surveying these patients indicate a preference for home treatment [27, 28]. However, limited documentation of health benefits according to site of care exists for patients with PIDD.

Results from the current study indicate that the incidence of lower respiratory tract infections was significantly lower in patients with PIDD who receive IGIV in a home setting compared with a hospital outpatient infusion center setting, independent of prophylactic antibiotic use. Assessment of the impact of the time interval post-infusion showed that significant differences in infection rates between sites of care were the largest in the time closest to the infusion; by the fourth week post-infusion, no significant differences in rates of infections were found between sites of care for either pneumonia or bronchitis. This suggests that perhaps the site of care itself may play a role, as the differences in infection rates for patients in the hospital outpatient infusion setting were significantly higher in the first few weeks after the infusion when the patient in that setting might be more likely to be exposed to pathogens. The fact that infection rates were not significantly different between sites of care by the fourth week after infusion suggests that the setting itself may be a factor. Given that health care expenditures for infections among patients with PIDD can be quite high, the economic impact of reducing infections by infusing in the home setting may be substantial [21]. This study extends the findings from a retrospective analysis by Luthra et al. [18] that showed that a significantly greater proportion of home-treated patients versus hospital-treated patients with PIDD adhered to the optimal dosing regimen of IGIV (*p* < 0.001). Luthra and colleagues further showed that patients infused at home incurred significantly lower IGIV-related administration costs (*p* < 0.001) and lower (but not significant) non-IGIV-related healthcare costs (*p* = 0.2128). The current analysis shows a significantly decreased incidence of lower respiratory infections among patients receiving home versus hospital IGIV treatment. This is a very important finding because long-term pulmonary disease that causes significant morbidity and premature mortality in PIDD patients is a consequence of recurrent lower respiratory tract infection. These data, therefore, support the notion that home-treated patients experience a health benefit as was also suggested by Luthra and colleagues [18].

Many PIDD patients with disorders of antibody production require lifelong IgG replacement [2, 3, 16]. Adherence to a treatment regime and avoidance of exposure to pathogens reduce the incidence of serious and recurrent infections that can be life-threatening to these patients [3]. Treatment is a time-consuming process that involves infusion and travel time every 3 to 4 weeks for patients receiving IGIV [12–15]. Our study indicates that home administration of IGIV, which ameliorates the time burden on patients and their families, is associated with a reduced frequency of bacterial respiratory tract infection. Although there are many factors that may, potentially, contribute to this benefit, it is possible that reduced exposure to nosocomial infections is one of those factors. The decision of where to administer IGIV, therefore, should also take into account the possible increased risk of exposure to pathogens in hospital settings.

Although this analysis was limited to IGIV recipients, and is subject to the caveats noted above, and there are no head-to-head studies comparing IGIV to IGSC, it is reasonable to consider the virtues of IGSC in the context of this observation. That is, home-based immunoglobulin treatment was associated with fewer lower respiratory tract infections than was treatment in hospital-based infusion centers. It is possible that this observation may be a partial explanation for the apparent patient preference for IGSC over IGIV. However, as patients and clinicians consider IgG treatment modalities, many are resistant to the frequent infusions and many needle sticks required for IGSC treatment.

However, several factors other than risk of infection and convenience influence the choice of site of care. These may include patient health, patient preference, venous access, quality of life issues, safety of the home environment, and support/network structure [16, 25]. Further research is warranted to understand which patient subpopulation would benefit the most from a home-based infusion setting.

### Limitations

A limitation of the current study is that an infection episode is expressed as a ≤7-day gap in claims. This duration may not be long enough for the types of infections analyzed and presented in this study. Although it is possible that an infection episode may last longer than 7 days, the authors judged that this was a reasonable timeframe during which a patient would seek healthcare services; however, this type of data was beyond the scope of the current analysis. There were differences in certain characteristics of patients between sites of care, notably, the age, CCI score, and number of inpatient hospitalizations in the year prior to IGIV therapy. While these variables were controlled in the regression analysis, it is possible that these differences may have affected the results. Differences in patient adherence between the sites of care could have also affected the results. However, due to the limitations of the data (i.e., the prescribed dose and actual dose administered were not available), it was not possible to assess patient adherence to their therapy in this analysis. Additionally, other possible confounders, such as lifestyle differences, socioeconomic differences, education status, and accessibility to medical resources, were not available for this analysis and therefore not included in the regression analyses.

## Conclusions

This study established that a home-based setting for IGIV infusions, compared with a hospital outpatient infusion center-based setting, was associated with significantly lower rates of bronchitis and pneumonia in patients with PIDD, after adjusting for age, gender, and illness severity. Risk of infection may therefore be an important factor in site of care selection. Considering the important limitations regarding this analysis, these findings may not be generalizable to other patient populations. Given the range of factors determining site of care, additional research may help elucidate which patient population would benefit most from home-based IGIV treatment.
